# Multiplex Zymography Captures Stage-specific Activity Profiles of Cathepsins K, L, and S in Human Breast, Lung, and Cervical Cancer

**DOI:** 10.1186/1479-5876-9-109

**Published:** 2011-07-14

**Authors:** Binbin Chen, Manu O Platt

**Affiliations:** 1 Wallace H. Coulter Department of Biomedical Engineering, Georgia Institute of Technology and Emory University, GA 30332, Atlanta, USA

## Abstract

**Background:**

Cathepsins K, L, and S are cysteine proteases upregulated in cancer and proteolyze extracellular matrix to facilitate metastasis, but difficulty distinguishing specific cathepsin activity in complex tissue extracts confounds scientific studies and employing them for use in clinical diagnoses. Here, we have developed multiplex cathepsin zymography to profile cathepsins K, L, and S activity in 10 μg human breast, lung, and cervical tumors by exploiting unique electrophoretic mobility and renaturation properties.

**Methods:**

Frozen breast, lung, and cervix cancer tissue lysates and normal organ tissue lysates from the same human patients were obtained (28 breast tissues, 23 lung tissues, and 23 cervix tissues), minced and homogenized prior to loading for cathepsin gelatin zymography to determine enzymatic activity.

**Results:**

Cleared bands of cathepsin activity were identified and validated in tumor extracts and detected organ- and stage-specific differences in activity. Cathepsin K was unique compared to cathepsins L and S. It was significantly higher for all cancers even at the earliest stage tested (stage I for lung and cervix (n = 6, p < .05), and stage II for breast; n = 6, p < .0001). Interestingly, cervical and breast tumor cathepsin activity was highest at the earliest stage we tested, stages I and II, respectively, and then were significantly lower at the latest stages tested (III and IV, respectively) (n = 6, p < 0.01 and p < 0.05), but lung cathepsin activity increased from one stage to the next (n = 6, p < .05). Using cathepsin K as a diagnostic biomarker for breast cancer detected with multiplex zymography, yielded 100% sensitivity and specificity for 20 breast tissue samples tested (10 normal; 10 tumor) in part due to the consistent absence of cathepsin K in normal breast tissue across all patients.

**Conclusions:**

To summarize, this sensitive assay provides quantitative outputs of cathepsins K, L, and S activities from mere micrograms of tissue and has potential use as a supplement to histological methods of clinical diagnoses of biopsied human tissue.

## Background

Tumor growth, migration, invasion and metastasis involves proteolytic activity, and the cathepsin family of cysteine proteases are proteases that have been implicated in each of these mechanisms, particularly cathepsins B, K, L, and S [1,2]. Cathepsin B is one of the more abundant cathepsins with lysosomal concentrations as high as one millimolar [3]. Much work has been done on the collagenolytic abilities of cathepsin B and its role in tumor metastasis [4,5] by degrading the basement membrane of tumor cells, but it has an occluding loop that makes its structure quite different from cathepsins K, L, and S [6].

Cathepsins K, L, and S are elastinolytic and collagenolytic cysteine proteases that share greater than 60% sequence homology [6], but the variable portions confer important differences in proteolytic activity and regulatory mechanisms. Cathepsin K is the most potent mammalian collagenase, capable of cleaving type I collagen in the native triple helix and in the telopeptide regions while other collagenases can only cleave at either one site or the other [7]. It was first thought to be exclusively expressed in osteoclasts, but there are a number of cell types that upregulate cathepsin K expression in cancer and other diseases [8-11]. Cathepsin L expression is increased in atherosclerosis and cancer as well and is secreted at sites of inflammation [12-15]. While cathepsins K and L prefer acidic environments for optimal activity, cathepsin S has the unique property of maintaining high elastinolytic activities at neutral pH and has been shown to be active in angiogenesis, lung cancer, and emphysema [16-18].

Cathepsin K has been particularly elusive in measuring its activity in cancer specimens. A number of studies have implicated cathepsin K expression in cancer progression and metastasis using cathepsin K inhibitors [19,20], mRNA analysis [21,22], and immunohistochemical labeling of normal and tumor sections [21-23], but the specific identification and quantification of the mature, active cathepsin K in these tumors has not been shown. These studies were important for implicating cathepsin K, but its transient nature and low levels of expression have made it difficult to specifically verify the mature form and detect its activity among a mix of other cathepsin family members. Radioactive, fluorescent, or biotinylated active-site probes have been coupled with blotting and histological protocols [24], and while they have increased sensitivity to visualize the mature form in a blot, they still do not provide measures of proteolytic activity, and cross-reactivity with other cathepsin family members confuse identification. Fluorogenic synthetic amino acid substrates have also been used to identify a single cathepsin member's activity above the others in complex cellular extracts and tissues [25,26], but due to the high sequence homology, the substrates are promiscuous. Even though one cathepsin may have a greater affinity and catalytic rate for a substrate, if another is present at higher concentrations, cross-reactivity will prevent an accurate measurement [22]. Similar specificity challenges exist for the use and development of small molecule inhibitors to cathepsin K [20].

Here, we describe multiplex cathepsin zymography, a technique that we recently developed that was capable of detecting cathepsin K activity down to femtomolar levels of recombinant enzyme and in macrophage derived osteoclasts [27]. Cathepsins L and S activity detection is not as sensitive, most likely due to cathepsin K being a much more powerful collagenase, but here, we have expanded its utility and demonstrated its multiplex capacity to detect cathepsins K, L, and S in cell or tissue preparations from breast, lung, and cervical tumors to profile cathepsin activity at increasing stages of cancer progression and provide a new tool to screen pathological specimens for previously undetectable cathepsin activity.

## Methods

### Human Tissues

Breast, lung, and cervix cancer tissue lysates and normal organ tissue lysates from the same patients were purchased from Protein Technologies Inc., San Diego, CA which is facilitated by Integrated Laboratory Services-Biotech (ILSbio). The original tumor and normal tissue specimens were collected from multiple hospitals. Tissue specimens were collected during the surgery process and immediately snap frozen with liquid nitrogen. ILSbio collected specimens under local Institutional Review Board approved protocols, ensuring each sample had patient consent for research purposes. 28 breast tissues, 23 lung tissues, and 23 cervix tissues were obtained (Table 1). Tumor samples were staged and graded by pathologists based on the American Joint Committee on Cancer (AJCC) Staging Manual [28]. Frozen tissues were minced and homogenized in cold modified RIPA buffer (PBS, 0.25% sodium deoxycholate, 0.1% SDS, 1 mM EDTA, 1 mM sodium fluoride, 1 mM sodium orthovanadate, 1 mM phenylmethanesulfonylfluoride, 1 μg/ml aprotinin, 1 μg/ml leupeptin, 1 μg/ml pepstatin A), and clarified by centrifugation. Protein concentrations of the lysates were normalized to 1 mg/ml.

**Table 1:** Patient Sample Characteristics

	Breast	Lung	Cervix
Normal	10	6	6
Stage I	Not Available	6	7
Stage II	6	6	6
Stage III	6	5	4
Stage IV	6	Not Available	Not Available
Age (Mean ± SD)	51.2 ± 5.6	56.5 ± 12.8	41.0 ± 11.0
Male/Female	0/28	6/17	0/23

### Gelatin zymography

Cathepsin zymography was performed as described previously [27]. Briefly, 5X non-reducing loading buffer (0.05% bromophenol blue, 10% SDS, 1.5 M Tris, 50% glycerol) was added to all samples prior to loading. Equal amounts of protein were resolved by 12.5% SDS-polyacrylamide gels containing 0.2% gelatin at 4°C. Gels were removed and enzymes renatured in 65 mM Tris buffer, pH 7.4 with 20% glycerol for 3 washes, 10 minutes each. Gels were then incubated in activity buffer (0.1 M sodium phosphate buffer, pH 6.0, 1 mM EDTA, and 2 mM DTT freshly added,) for 30 minutes at room temperature. Then this activity buffer was exchanged for fresh activity buffer and incubated for 18-24 hours (overnight) incubation at 37°C. The gels were rinsed twice with deionized water and incubated for one hour in Coomassie stain (10% acetic acid, 25% isopropanol, 4.5% Coomassie Blue) followed by destaining (10% isopropanol and 10% acetic acid ). Gels were scanned using an Imagequant 4010 (GE Healthcare). Images were inverted in Adobe Photoshop and densitometry performed using Scion Image.

MMP zymography was similar except the enzymes were renatured in 2.5% Triton-X and incubated in 50 mM Tris-HCl pH 7.4, 10 mM calcium chloride, 50 mM sodium chloride, 0.05% Triton-X assay buffer overnight. Gels were imaged using an Imagequant 4010 (GE Healthcare, Waukesha, WI). Images were inverted in Adobe Photoshop and densitometry was performed using Scion Image. Representative zymograms shown here have had the levels adjusted for the entire gel image to improve print viewing clarity. All human recombinant cathepsins were from Enzo Life Sciences (Plymouth Meeting, PA). Human cathepsins K and S were expressed in insect cells, human cathepsin V was expressed in NSO cells, and human cathepsin L was isolated from liver.

### Western blotting

SDS-PAGE was performed, and protein was transferred to a nitrocellulose membrane (Bio-Rad) then probed with monoclonal anti-cathepsin K antibody clone 182-12G5 (Millipore, Billerica, MA) or anti-cathepsin L, or S antibodies (R&D Biosystems, Minneapolis, MN). Secondary donkey anti-mouse or anti-goat antibodies conjugated to an infrared fluorophore (Rockland, Gilbertsville, PA) were used to image protein with a Li-Cor Odyssey scanner (Lincoln, Nebraska).

### Statistical Analysis

Results are shown as mean ± SEM of normal and tumor groups. Student's unpaired t-test was used to evaluate statistical significance between two result groups. Values of p < 0.05 were considered statistically significant. Sensitivity, specificity, and likelihood ratio of the corresponding protease biomarker were calculated across a range of threshold values with Matlab (Mathworks). To determine the optimal threshold value that would maximize sensitivity and specificity, we input the range of values from zero to the larger value of either the maximum protease value measured in normal specimens or the minimum value measured in the cancer specimens. Threshold window index was calculated according to the following formula:

## Results

### Multiplex cathepsin zymography detects mature cathepsins K, L, and S activity

**Figure 1:**
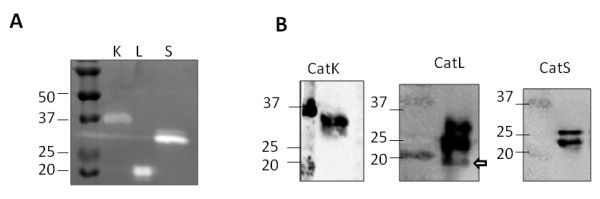
**Multiplex cathepsin zymography detects mature cathepsin K, L, and S activity at distinct migration distances**. **A) **Human recombinant cathepsins K (1 ng), L (50 ng), and S (20 ng) were loaded for cathepsin gelatin zymography (left) and **B) **Western blotting. Arrow is used to indicate the zymographically active band on cathepsin L blot.

Mature cathepsins K, L, and S were loaded for cathepsin zymography and parallel samples were loaded for Western blotting to first determine if the zymographically active bands of cathepsins K, L, and S would appear at different electrophoretic migration distances. Different amounts of each cathepsin were loaded to produce clear bands in the zymogram as they have different limits of detection by the zymography assay (data not shown). Cathepsins K, L, and S (1, 50, and 20 ng, respectively) all appeared as zymographically active bands at distinct molecular weights (Figure 1); mature cathepsin K band appeared near the 37 kDa size, cathepsin L at 21 kDa, and cathepsin S near 25-27 kDa (Figure 1A). Migration distances (or apparent molecular weights) were compared with the Western blots in figure 1B to verify the identity of each band. The immunodetected cathepsin K band is near 37 kDa, cathepsin S exhibited two bands near 25 kDa, and the cathepsin L protein was detected at three sizes, but only the smallest of the three immunodetected bands was zymographically active (Figure 1B).

### Cathepsin zymography detects 50-fold increased cathepsin K activity in breast cancer specimens

**Figure 2:**
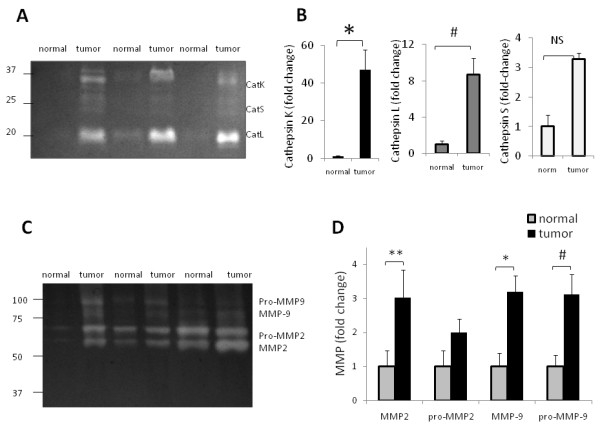
**Cathepsins K, L, and S activity detection in human breast tissue**. **A) **10 μg of normal and tumor breast tissue from patient biopsies were loaded for zymography. Cathepsin K band is visible at 37 kD, cathepsin L at 21 kD, and cathepsin S at 25 kD. Representative zymogram is shown and cropped for clarity. **B) **Cathepsin activities were quantified with densitometry of each band on the gel. **C) **The same samples were loaded for MMP zymography. A representative MMP zymogram is shown and cropped for clarity. **D) **Pro- and mature MMP-9 and MMP-2 activities were quantified by band densitometry. All values are fold change of tumor compared to normal (n = 10, #p < .005, *p < .002, ** p < .05).

Once it was determined that cathepsins K, L, and S could be detected with cathepsin zymography, we tested the hypothesis that cathepsin K activity would be significantly increased in breast cancer tissue compared to normal tissue, and that zymography would detect these differences. Equal amounts of breast tissue protein (10 μg) were loaded for cathepsin zymography and quantified by densitometry (Figure 2A). In these ten patient-matched breast cancer tissue specimens tested, cathepsin K activity was 50-fold higher than the activity in normal breast tissue (n = 10, p < .002), cathepsin L was 9-fold higher (n = 10, p < .005), and cathepsin S was 3-fold higher but not statistically significant (Figure 2B). Patient and tumor information is given in Table 1.

Matrix metalloproteinases (MMPs) are another family of proteases that are metal dependent endopeptidases implicated in cancer development and metastasis [29,30]. MMP-2 and -9 are among the most studied members and gelatin zymography identifies their activity, but the assay buffer for optimal activity is different pH and composition than that for cathepsins as described here. Incubation of cathepsin zymography gels in acidic conditions drastically reduces the activity of MMPs and serine proteases, and the addition of EDTA, a calcium and zinc chelator, to the assay buffer also prevents activation of the calcium dependent calpains and MMPs to promote cathepsin selectivity. To determine if MMP activity was as upregulated in tumor specimens as the cathepsin activity, the same tissue specimens from Figure 2A were loaded for MMP zymography. Tumor MMP-2 and -9 activities were only 2-3 fold greater than normal tissue (Figure 2 C, D p < .05); much less than the 50- and 9-fold increases found in the cathepsin K and L zymograms, respectively.

### Stage-specific differences in cathepsins K, L, and S in human breast cancer

**Figure 3:**
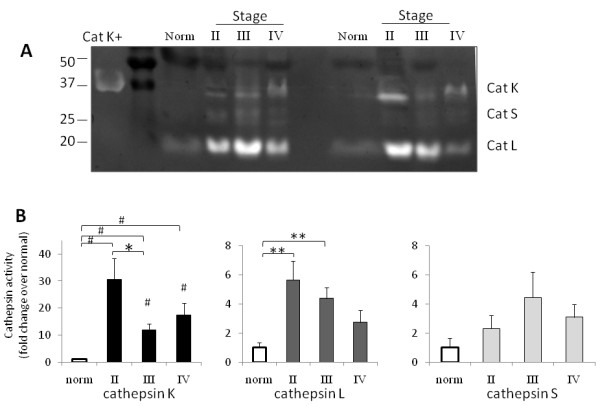
**Stage-specific differences in cathepsins K, L, and S in human breast cancer**. **A) **10 μg of total protein from breast tissues from stage II-IV from normal and cancer breast tissues were loaded for multiplex cathepsin zymography, and a representative zymogram is shown and cropped for clarity. **B) **Cathepsins K, L, and S activities were quantified by band densitometry (n = 5, *p < 0.05, **p < 0.01, #p < 0.0001).

We next wanted to determine any stage specific differences in breast cancer cathepsin activity using this cathepsin zymography assay. At least five different specimens each of stages II, III, and IV breast tumor tissue (as determined by the TNM staging system according to AJCC Staging Manual) and normal tissues were obtained and loaded for cathepsin zymography. Stage I and premalignant breast tissue samples were unavailable to us. Cathepsin activity peaked at stage II and declined through stages III and IV (Figure 3A, B). It is important to note that for cathepsin K, tumor activity at all stages tested in these samples was significantly higher than the normal breast tissue activity by 10- to 30-fold (n = 5-8, *p < 0.05, **p < 0.01, #p < 0.0001) (Figure 3B). Cathepsin L activity was significantly higher than normal at stages II and III (n = 6, p < .05), but not at stage IV, and due to variability among the five samples tested at each stage, there was no significant increase in cathepsin S activity (n = 6).

### Utility of cathepsin K zymography as a clinical biomarker assay for breast cancer detection

**Figure 4:**
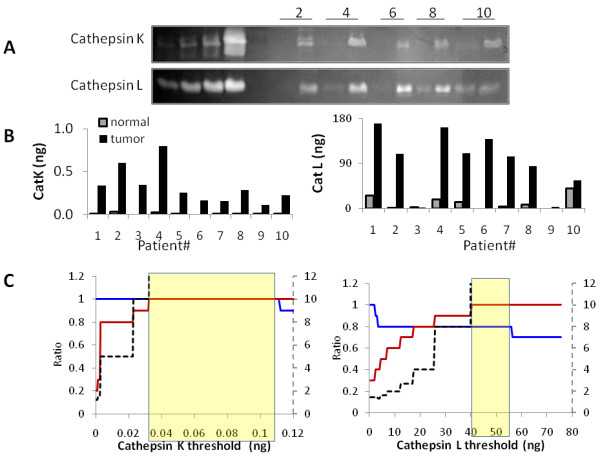
**Cathepsin K zymography potential as a clinical diagnostic tool for breast cancer . A) **Normal and tumor breast tissue zymograms were compared for patient-to-patient variation. Standard dose curves of recombinant cathepsin K (0.2, 0.5, 1, and 5 ng) and cathepsin L (45, 220, 450, and 900 ng) were loaded per gel for quantifying absolute quantities of cathepsins K and L. **B) **Cathepsins K and L activity were quantified with densitometry and compared to the standard curve generated to semi-quantitatively determine nanograms of active enzyme. **C) **Sensitivity (blue line), specificity (red line), and likelihood ratio (dotted black line) were calculated over a range of values to identify an optimal threshold value for cathepsins K and L that would distinguish normal samples from tumor samples. Yellow boxes outline the region of maximal likelihood ratio.

Patient-to-patient variation in cathepsin K and L activity was assessed to determine if a threshold value of cathepsin K activity could be set that, once crossed would indicate a positive cancer specimen, (Figure 4A). Absolute amounts of cathepsin K activity per 10 μg breast tissue protein was determined by loading increasing doses of recombinant cathepsins K and L in the same gel as the breast cancer and normal specimens to generate a standard curve to which the specimen signal could be fit. Across all ten normal specimens, cathepsin K measurements were between 0 and 0.03 ng per 10 μg of tissue protein (Figure 4B). For the cancer samples, the range of values of cathepsin K were from 0.112 ng to 0.8 ng per 10 μg of tissue protein (Figure 4B), up to almost two orders of magnitude higher than any of the normal specimens. The patient variability for cathepsin L is shown as well but was not as consistently low for the normal specimens or as consistently high for the tumor specimens (Figure 4B).

Sensitivity and specificity analyses were performed to quantify the probability of a sample being correctly or incorrectly diagnosed by zymography for cathepsins K and L. Likelihood ratios were calculated to select the maximum sensitivity and specificity for each protease tested, and the ranges of values over which the likelihood ratio is maximized are highlighted by the yellow box (Figure 4C). Cathepsin K was the only enzyme of those tested that reached 100% sensitivity and 100% specificity across the twenty breast tissue specimens of this study. Cathepsin L sensitivity and specificity values were 80% and 100%, respectively (Figure 4). MMP-2 sensitivity and specificity were 60% and 90%, and MMP-9 had values of 80% and 90% (Table 2, Additional File 1). A threshold window index was calculated for each protease as the ratio of the difference in the range of values that maximize likelihood ratio to the maximum potential threshold value. The results are shown in Table 2 with cathepsin K having the largest threshold window index (72%) to provide this maximum sensitivity and specificity.

**Table 2:** Range of threshold values at maximal likelihood ratio and associated sensitivity and specificity values for each protease tested

Range	Enzyme	Index	Sensitivity	Specificity
.03-0.11 ng	Cathepsin K	72%	100%	100%
40-55 ng	Cathepsin L	27%	100%	80%
4639-5009 AU	MMP-9	7%	80%	90%
6447-7063 AU	proMMP-9	9%	70%	90%
5334-5872 AU	MMP-2	9%	60%	90%
4762-5541 AU	proMMP-2	14%	80%	80%

### Cathepsins K, L, and S activity profiles in human lung cancer

**Figure 5:**
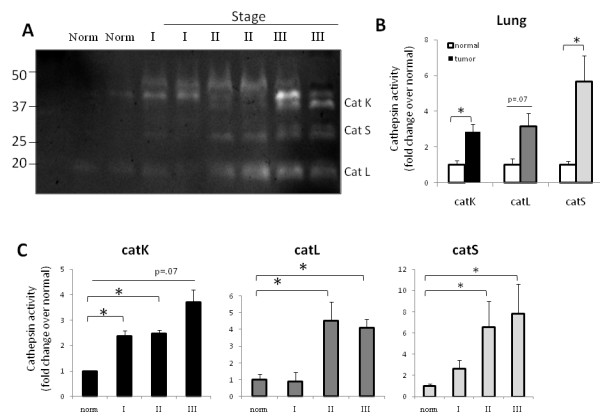
**Cathepsins K, L, and S activity profiles in human lung cancer**. **A) **Different stages (I, II, and III) of lung cancer and normal lung tissues were obtained and prepared as described. 10 μg of protein were loaded for multiplex cathepsin zymography, and a representative zymogram is shown and cropped for clarity. **B) **Cathepsin K, L, and S activities were quantified by densitometry. Cathepsin activity from 24 samples (18 cancer and 6 normal) comparing normal to tumor is shown. **C) **Comparisons of cathepsins K, L, and S activity changes at different stages of tumor progression (n = 4-6, *p < 0.05).

With successful detection of mature cathepsins K, L, and S in human breast cancer tissue, other types of tumors were investigated to establish broader utility of this assay as a screen for multiple cathepsins in one tissue specimen. Cathepsin K had been previously identified immunohistochemically in lung tumor specimens [31,32], but the active mature enzyme had not been measured. Normal and tumor lung tissue specimens from stages I, II, and III were obtained, and loaded for cathepsin zymography (Figure 5A). Lung tumor specimens had a statistically significant increase over normal tissue in cathepsin K (2-3 fold) and cathepsin S (5-6 fold), but not for cathepsin L (~2-3 fold, p = .07) across all stages tested (Figure 5B). Comparisons were then made between stages to measure lung tumor stage-specific differences in cathepsin activity. Cathepsins K, L, and S activity all increased with lung tumor stage, but most notably, only cathepsin K showed a statistically significant increase in activity as early as stage I (Figure 5C). Cathepsins L and S were significantly higher than normal by stages II and III for the lung tumor specimens tested (Figure 5C).

### Increased cathepsin K in human cervical cancer specimens

**Figure 6:**
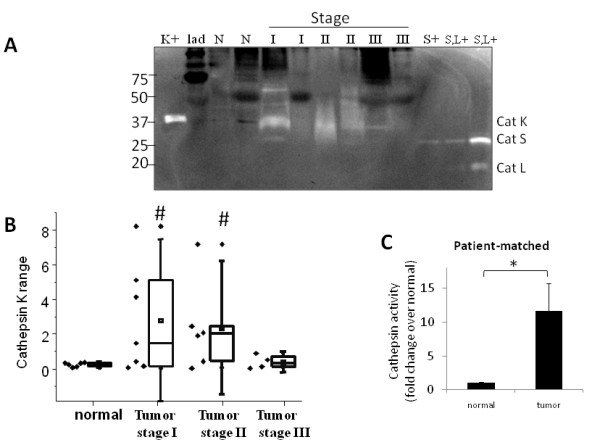
**Increased cathepsin K in human cervical cancer specimens**. **A) **Tumor tissues from stages I-III cervical cancer and normal tissues were obtained and prepared as described. 10 μg of protein were loaded for multiplex cathepsin zymography, and a representative zymogram with protein ladder (lad), cathepsin K, L and S positive controls is shown and cropped for clarity. **B) **Cathepsins K, L, and S activities were quantified by band densitometry and represented by the box and whisker plot shown to exhibit patient to patient variability. For box and whisker plots, the top and bottom of the box represent the 75^th ^and 25^th ^quartile, and whiskers +1.5 SD and -1.5 SD, respectively (n = 4-7, #p < 0.05 compared to normal). **C) **Patient-matched comparisons of normal to tumor cervical tissue cathepsin K activity yielded an increase of ~12 fold (n = 5, *p < 0.05).

Multiple proteases have been shown to be related to cervical cancer development [33,34], but there have been no reports of cathepsin K involvement. Normal and tumor cervical tissue specimens from stages I, II, and III were obtained and loaded for multiplex cathepsin zymography (Figure 6A). Human recombinant cathepsins K, L, and S positive controls were loaded as well to confirm cervical cathepsin identity. The dominant cathepsin active in the zymography of cervical tumor extracts was cathepsin K (Figure 6A); cathepsin K activity was highest at stages I and II, but not significantly different in stage III cervical tumors (Figure 6B).

Cervical tumor specimens' cathepsin K activity displayed a wide range of patient-to-patient variability, as seen in the box-whisker plot, and, as a result, comparisons of all normal samples to all tumor samples was not statistically significant. However, there were significant differences determined between normal cervical tissue and stage I and stage II cervical cancer tissue, but not that of stage III (Figure 6B). To remove patient-to-patient variability as a confounding factor, we analyzed the combined data using only paired normal and malignant cervical tissue from the same patient (n = 5). In figure 6C, cathepsin K activity in the cervical tumor is significantly increased by 10-fold for an individual above her own basal normal tissue activity levels (n = 5, p < .05).

### Comparison of cathepsin activity among different organs

**Figure 7:**
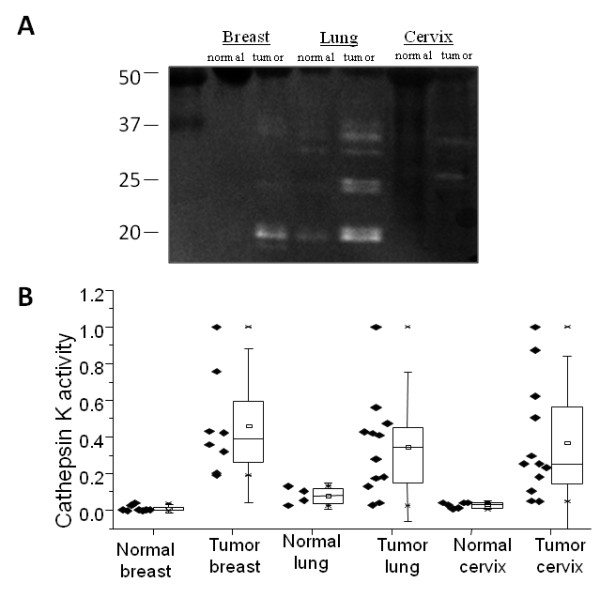
**Comparisons of different organ samples**. **A) **Normal and tissue samples from the three organs (breast, lung and cervix) were obtained and prepared as described. 10 μg of protein were loaded for multiplex cathepsin zymography, and a representative zymogram is shown and cropped for clarity. **B) **Cathepsin K data was normalized by maximum cathepsin K activity value for each organ and represented by the box and whisker plot. For box and whisker plots, the top and bottom of the box represent the 75^th ^and 25^th ^quartile, and whiskers +1.5 SD and -1.5 SD, respectively.

To compare cathepsins K, L, and S activity across all three tissues tested and observe any differences in normal baseline signatures as well as cancer-mediated increases, 10 μg of protein from each organ, normal and tumor, were loaded into one zymogram (Figure 7A). Lung baseline and tumor activity was higher than both breast and cervix. In order to quantify differences in organ specific increases in cathepsin activity from normal to tumor, cathepsin activity was normalized to the maximum signal for each organ and presented as box-whisker plots (Figure 7B). For breast, lung, and cervix tissue, the tumor specimens showed increased cathepsin K activity, with minimal to no detection in normal tissue (Figure 7B). Cathepsin K activity was elevated in the tumor samples of all three cancers tested: breast, lung, and cervix (Figure 7B).

## Discussion

Multiplex zymography's utility as a supplemental screening tool of pathological specimens was effectively shown here to profile cathepsin K, L, and S activities in breast, lung, and cervical tissue at three different stages of tumor progression. This matrix of information was captured by this one assay after clinical grading of the biopsied tissue indicating that quantitative comparisons with cathepsin zymography can supplement the gold standard histological methods of determining whether biopsied tissue is cancerous or not.

Differences in organ and tissue structure or predominant extracellular matrix (ECM) components may be responsible for the differences in cathepsin K, L, and S activity profiles between breast, lung, and cervical tissue and changes to these profiles as the cancer stage increased. Ductal carcinoma breast cancers arise in the inner layer of mammary duct in the columnar epithelium that lines it, and are surrounded by lobes, stromata, and adipose tissues. Squamous cell carcinoma of the cervix starts in the epithelium of cervix and invades into the underlying stroma. Lung tumors, however, that mainly arise in the bronchi, are surrounded by hyaline cartilage, a tough connective tissue made of high density of collagen II and is also in a much more dynamic environment as the diaphragm contracts and relaxes during breathing. ECM proteins [15], mechanical forces [14,35], native and infiltrating cell types [36,37], and cell transformation [2,38] have all been linked to upregulation of different cathepsin family members and all may contribute to the organ-specific differences seen here. Cathepsin secreting alveolar macrophages are regularly present in the lungs [39] and may contribute to the higher baseline of cathepsin activities in lung tissues compared to breast and cervical tissues (Figure 7).

Of the three types of cancer and the three cathepsins studied, cathepsin K in breast tissue was especially unique in that its activity was binary: off in normal tissue and on in cancerous tissue. Cathepsin K in breast tissue had the lowest variability and consistently low baseline for cathepsin K activity in the normal tissue, compared to breast, lung, and cervical cancer tissue (Figure 4). This suggests that across a number of patients, the background and basal activity is low for healthy, noncancerous breast tissue tempting consideration of using cathepsin zymography for clinical detection of breast cancer. A potential clinical workflow closely resembles that of the one followed for these specimens prior to reaching our lab for examination: 1) lump detection by self exam or mammogram, 2) visit to doctor, 3) biopsy of small piece of tissue, 4) histological assessment performed by pathologist, and 5) zymography from 10 μg biopsied tissue protein and comparison to threshold value. A greater number of clinical specimens will need to be assessed to determine efficacy of zymography in practice, prior to clinical grading, but the results shown here with 100% sensitivity and 100% specificity are promising.

Higher patient-to-patient variability of cathepsin levels within lung and cervical cancer specimens tested here may be due to the source of the tumorigenicity for each organ. National Cancer Institute reports that smoking is the leading cause of lung cancer deaths: 90 percent for men and 80 percent for women (NCI 2010), and differences in smoking habits and tobacco delivery methods may be the cause of the variability detected by this assay. Despite the variability within a stage of lung cancer, there was still a statistically significant increase in cathepsin activity compared to normal. Again, cathepsin K activity stood out as being significantly upregulated even at stage I of lung cancer, where cathepsin L and S did not reach this point until stage II (Figure 5C). Unique upregulation of cathepsin K in lung tumors also seems to be a candidate biomarker for early confirmation of lung cancer detection.

Human papilloma virus (HPV) infection is a leading cause of cervical cancer [33] and has been shown to influence cathepsin levels in mouse models of cervical cancer [40]. Different strains of HPV have different amplification and oncogenicity [33], and may be reflected in the variations of the human cervical cancer results (Figure 6B). We were not aware of HPV status of any of the specimens. However, there was still a significant increase in cathepsin K activity in cancerous cervical tissue when compared to normal cervical tissue from that same patient (Figure 6C). This patient-matched data for six women corroborates evidence that cervical tumors express greater levels of cathepsin K activity once the patient variability factor was removed.

Pap smears are routinely performed to screen for cervical cancer. Small samples of cervical tissue are biopsied and clinical and pathological grading of the histology is performed to observe any abnormal cells in the samples. Given the results shown here with a 10-fold increase in cathepsin K activity detected from 10 μg of tissue protein, cathepsin zymography may serve as a supplemental biomarker to aid the assessment of inconclusive Pap smear results. Again, more clinical samples will need to be tested to verify its utility, but cathepsin K also presented an on/off activity in cancer vs. normal cervical tissue, similarly to breast tissue.

Cervical and breast cancer cathepsin activity peaked at the earliest stage we tested, stages I and II, respectively, and then were significantly less at the latest stage tested. This non-intuitive change in cathepsin activity in the primary tumor has not previously been shown. Tumor cell heterogeneity may provide one possible explanation. Our hypothesis is that the more metastatically inclined cells in the primary tumor are producing more cathepsin proteases to facilitate departure from the primary tumor site; when they leave the primary tumor, the source of the proteolytic activity leaves as well. This finding may guide improved stage specific treatments for tumors and indicate more rigorous protease inhibition strategies at primary tumors to block the earliest steps of metastasis.

It is important to note this zymography assay samples the entire tumor, not just the tumor cells. Therefore, any tumor associated macrophages, blood vessels, white blood cells, or any other infiltrating cells with cathepsin activity will be captured in that tissue extract. Aggressive tumor cells are able to recruit the surrounding stromal cells to enhance tumor growth. Tumor associated macrophages expressing cathepsins were shown to organize around the tumor edge at later stages in a pancreatic cancer animal model [36], indicating that there is cellular recruitment and organization that may promote metastasis. Their combined activities contribute to tumor metastatic potential and this zymography assay corporately analyzes their cathepsin activity profile. This raises the issue of whether cathepsin K zymography will be able to differentiate cancer from benign tissue hypertrophies and inflammatory diseases. As macrophages actively participate in most of immune responses, elevated cathepsin activities at inflammatory situations are theoretically possible and need to be further tested with clinical samples. Fluorescent activity based probes (ABP) in tissue sections provide incredible resolution of cathepsin activity [41] and, used in conjunction with zymography, can provide a two-prong identification approach: ABP on tissue slices with cell-specific immunohistochemical labelling can identify cell types producing cathepsins, and zymography can identify the type and quantity of cathepsin being produced.

## Conclusions

Overall, the application of multiplex cathepsin zymography to breast, lung, and cervical cancer specimens have highlighted the unique upregulation of cathepsin K in all three of these cancers tested and even in the earliest stages measured. Altogether, this elevates cathepsin K potential to be a cancer biomarker for breast, lung, and cervical cancer, but more broadly, highlights its potential to be a biomarker for other types of cancer that have not previously been investigated. Histological studies benefit from quantitative, visual confirmation offered by cathepsin zymography using just a small piece of the biopsied tissue. As an added benefit to researchers, this assay does not require antibodies, which expands its application to other species, including the numerous mouse models of cancer that have been developed. Lack of antibodies also significantly reduces cost compared to immunobased methods such as ELISA, Western blotting, and immunohistochemistry, as well as minimizing concerns of nonspecific antibody binding and pro-cathepsin detection interference. Employing such multiplex technologies, that can screen samples inexpensively, will provide a broader net to catch new biomarkers and etiological agents to direct investigation into previously untested mechanisms and inhibitory targets.

## Competing interests

The authors declare that they have no competing interests.

## Authors' contributions

BC participated in the design of the study, conducted experiments, performed statistical analysis, and helped draft the manuscript. MOP conceived of the study and participated in its design and coordination, conducted experiments, and helped to draft the manuscript. All authors read and approved the final manuscript.

## Supplementary Material

Additional files 1 **Diagnostic performance of MMP-2 and MMP-9 for breast cancer**. Sensitivity (blue line), specificity (red line), and likelihood ratio (dotted black line) were calculated and plotted over a range of values to identify an optimal threshold value for MMP-2 and MMP-9 that would distinguish normal samples from tumor samples. Yellow boxes outline the region of maximal likelihood ratio. Click here for file
