# Supplementary figures and images for: Multiplex Zymography Captures Stage-specific Activity Profiles of Cathepsins K, L, and S in Human Breast, Lung, and Cervical Cancer
Source: J Transl Med. 2011 Jul 14;9:109. doi: 10.1186/1479-5876-9-109 (PMC3146840; doi:10.1186/1479-5876-9-109)

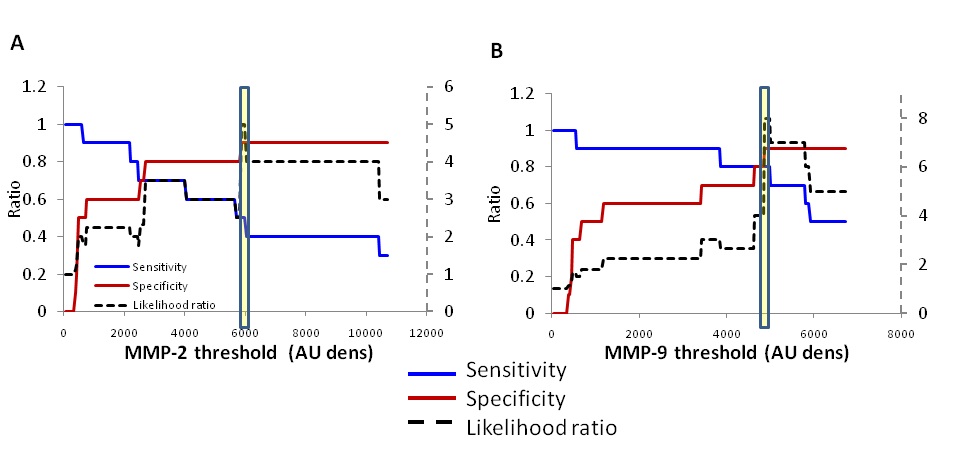

Supplement: Additional files 1 — Diagnostic performance of MMP-2 and MMP-9 for breast cancer. Sensitivity (blue line), specificity (red line), and likelihood ratio (dotted black line) were calculated and plotted over a range of values to identify an optimal threshold value for MMP-2 and MMP-9 that would distinguish normal samples from tumor samples. Yellow boxes outline the region of maximal likelihood ratio. [file 1479-5876-9-109-S1.TIFF]
